# Anaplastic Large Cell Lymphoma Related to Breast Implant Presenting as a Solid mass: A Case Report

**DOI:** 10.1155/cris/1393257

**Published:** 2025-11-24

**Authors:** Nadia Gabriele Walsh, Sandra Hembrecht, Sally McGrath, Deirdre Duke, Arnold Hill, John Quinn, Neasa Ni Mhuircheartaigh, Laura McKenna, Colm Power

**Affiliations:** ^1^Department of Surgery, Royal College of Surgeons in Ireland, County Dublin, Dublin, Ireland; ^2^Beaumont Breast Centre, Beaumont Hospital, County Dublin, Dublin, Ireland; ^3^Histopathology Department, Beaumont Hospital, County Dublin, Dublin, Ireland; ^4^Haematology Department, Beaumont Hospital, County Dublin, Dublin, Ireland

## Abstract

**Introduction:**

Breast implant associated anaplastic large cell lymphoma (BIA-ALCL) is an uncommon form of non-Hodgkin's T-cell lymphoma associated with textured breast implants and tissue expanders.

**Case Presentation:**

A 46-year-old female presented with a 1-week history of a firm lump in the upper inner quadrant of her right breast. She had a history of augmentation mammoplasty with textured implants 8 years prior. Ultrasound guided biopsy of the 15 mm × 18 mm mass confirmed a diagnosis of BIA-ALCL on histopathology. Pre-operative staging with PET CT showed a peri-implant nodule with avid nodules within and deep to the pectoralis muscle with no evidence of distant metastases. Bilateral en bloc removal of the breast implants, capsulectomy (including palpably involved pectoralis major and minor muscles) and right axillary dissection were performed. Final histopathology confirmed BIA-ALCL, pT4N0, with clear margins. Post-operative PET-CT demonstrated complete excision of local disease, however, a new FDG-avid right internal mammary node was identified, which increased in size and avidity on follow up imaging. Mediastinoscopy with core biopsy were performed and histopathological features were consistent with metastatic cells (BIA-ALCL). Patient subsequently completed six cycles of adjuvant chemotherapy with evidence of interval response on imaging. To date, patient remains in complete clinical and radiological remission and the expected duration of follow up is 5 years.

**Conclusions:**

BIA-ALCL poses a significant challenge due to increasing use of implants for reconstructive and cosmetic procedures. Patients most commonly present with peri-implant fluid collections but palpable masses, capsular contracture and lymphadenopathy are also commonly seen. Diagnosis involves ultrasound and histopathological analysis of fluid or tissue with CD30 immunohistochemistry and staging with PET-CT. Patient education and a multidisciplinary team approach allow for timely diagnosis and complete surgical excision, which are key for a good prognosis. Clinical and radiological surveillance detect early recurrence and assess need for adjuvant therapy.

## 1. Introduction

Breast implant associated anaplastic large cell lymphoma (BIA-ALCL) is an uncommon form of non-Hodgkin's T-cell lymphoma associated with textured breast implants and tissue expanders [1]. It was first reported by Keech and Creech [2] and recognised as a distinct cancer by the World Health Organisation in 2016 [3].

The current reported incidence worldwide is very low with the patient-specific cumulative risk within the U.S. market ranging from 1.79 per 1000 (1:559) to 2.82 per 1000 (1:355) patients with a textured implant [4]. Increased use of implants for cosmetic and reconstructive reasons predicts an increased prevalence [5]. As of August 5th, 2024, the American Society of Plastic Surgeons reported approximately 1602 cases of BIA-ALCL worldwide [6].

Chronic inflammation seems to play a significant role in the development of this iatrogenic malignancy, especially with textured implants, even though its aetiology is not fully understood [7].

We would like to present the case study of a patient diagnosed with BIA-ALCL at our institution. An abstract about this case was previously presented as a poster presentation in the Sylvester O'Halloran Perioperative Symposium in Limerick, Ireland [8].

## 2. Case Presentation

A 46-year-old female, presented to the Beaumont Breast Centre, Dublin, Ireland, with a 1-week history of a firm, mobile lump in the upper inner quadrant of her right breast measuring approximately 2 cm clinically as well as palpable axillary lymph nodes. She had a history of augmentation mammoplasty with textured implants (Allergan Natrelle INSPIRA, style TRF) performed in Ireland 8 years prior. She had prior gastric band surgery and had no personal or family history of breast malignancy. Her regular medications included hormone replacement therapy and a proton pump inhibitor. She smoked occasionally.

**Figure 1:**
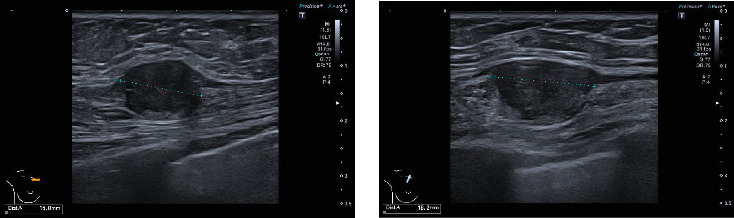
Breast ultrasound images demonstrating the original 15 mm × 18 mm mass on the upper right chest wall area which was originally biopsied (27/04/2023).

On clinical examination, a 2 cm firm mobile lump was identified at the upper inner quadrant of patient's right breast, S3 (indeterminate, probably benign) [9], as well as multiple palpable right axillary lymph nodes. Following clinical examination, patient underwent bilateral mammogram with tomosynthesis including implant displaced views and targeted ultrasound of right breast and axilla on the same day. Mammogram showed bilateral sub glandular implants. The breasts were normal bilaterally. The clinical area of concern was high on the chest wall outside the mammogram field. Ultrasound demonstrated a discrete 15 mm × 18 mm solid mass in the upper right chest wall (Figure 1). No axillary adenopathy was identified on imaging. A score of R4 (moderately suspicious) [9, 10] was given, and an U.S.-guided core biopsy was performed using a 14-gauge tru-cut core biopsy needle.

A contrast enhanced breast magnetic resonance imaging (MRI) was performed for further evaluation. This identified a focal area of enhancement just superior to right peri-implant capsule, anterior to the pectoralis muscle. There was low volume fluid surrounding the right implant within the capsule; however, the implant was intact and there was no axillary or internal mammary lymphadenopathy on imaging.

**Figure 2:**
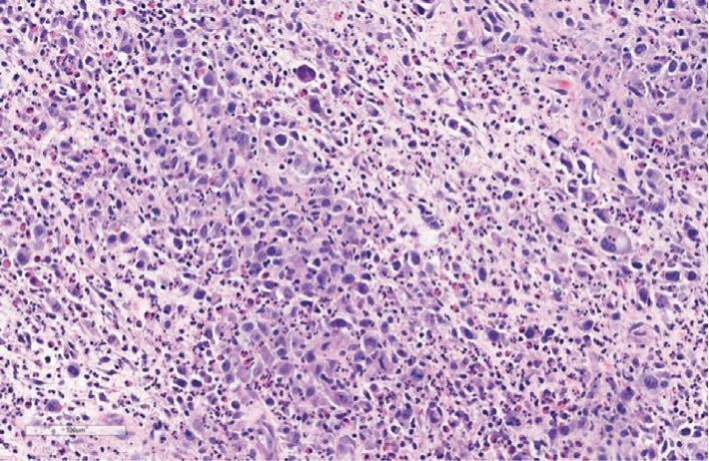
H&E image of original core biopsy, showing large atypical malignant cells diffusely spread in an inflammatory background.

**Figure 3:**
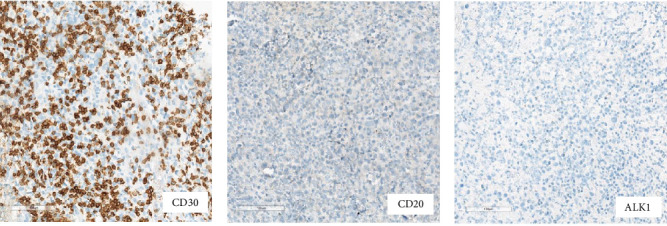
Image of tumour in original biopsy, stained with CD30, CD 20 and ALK-1, respectively.

A core biopsy was undertaken, demonstrating an inflammatory infiltrate with admixed large, atypical cells (Figure 2) which were immunoreactive for CD30 (Figure 3) and CD3. ALK1, CD20 and PAX5 were negative consistent with ALCL (Figure 3). The clinical presentation, imaging and ALK1 negativity supported a diagnosis of BIA-ALCL, rather than primary systemic disease. A clonal T-cell receptor gamma chain gene rearrangement was identified by PCR on the subsequent resection specimen.

**Figure 4:**
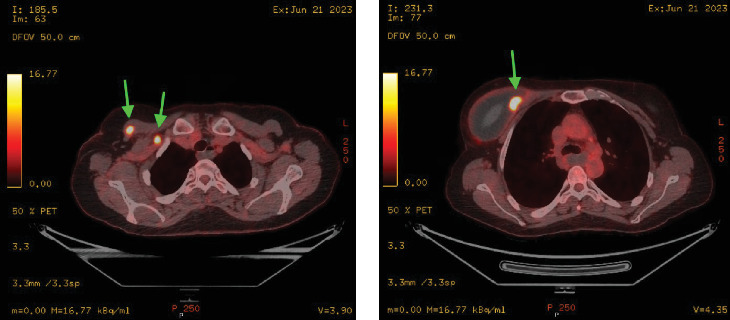
PET-CT, fused axial series, images 63 and 77, respectively (21/06/23). Images show biopsy-proven BIA-ALCL with abnormal avid sub-pectoral nodes (green arrows).

**Figure 5:**
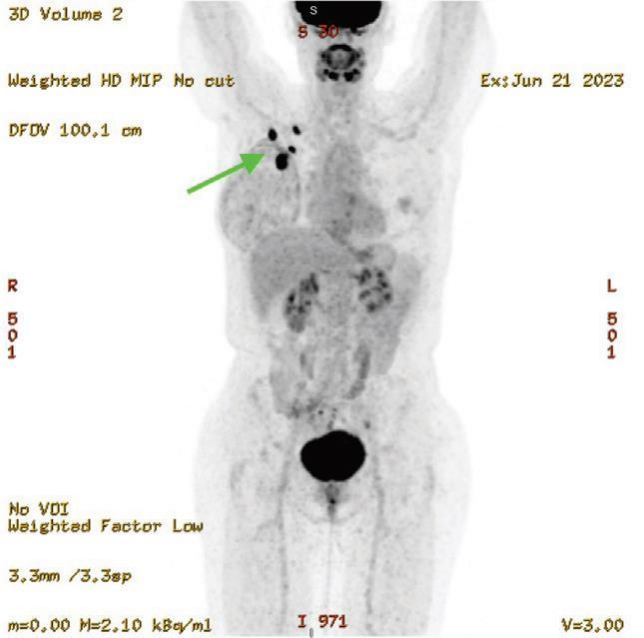
PET-CT, MIP (21/06/23). Image shows biopsy-proven BIA-ALCL with abnormal avid sub-pectoral nodes (green arrow).

Staging was completed with positron emission tomography (PET-CT) which demonstrated FDG avid periprosthetic nodule in the right breast, as well as FDG avid right pectoralis major intramuscular nodules and FDG avid right subpectoral node (Figures 4 and 5).

After multidisciplinary meeting consensus, this patient underwent surgical treatment with bilateral breast capsulectomy, removal of breast implants and right axillary nodal dissection. Axillary dissection was performed due to the presence of multiple clinically palpable lymph nodes, even though negative on imaging. The capsule extended deeply into pectoralis major and minor muscles. All palpable disease was excised including a substantial amount of pectoralis major and minor.

**Figure 6:**
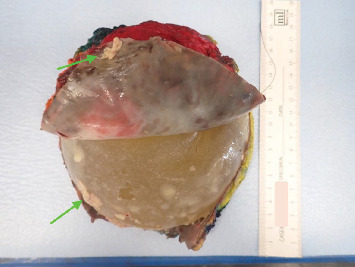
Gross image of capsulectomy specimen, showing the capsule peeled back from the underlying implant, with multiple tumour foci (green arrows).

**Figure 7:**
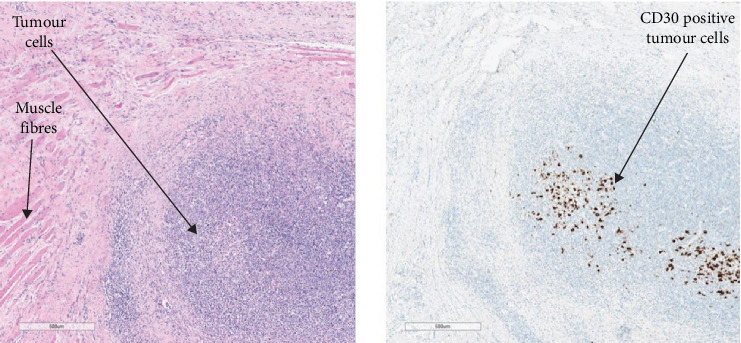
Capsulectomy specimen showing site of muscle invasion, respectively: 40x H&E, showing tumour and adjacent muscle fibres (arrows) and 40x image of the same area stained for CD30 and demonstrating the CD30 positive malignant cells (arrow).

The right capsulectomy specimen contained multiple tumour masses within the capsule cavity, some of which were free floating, with others adherent to the breast implant and the fibrous capsule (Figure 6). It focally extended through the capsule into muscle (Figure 7). A total of 14 lymph nodes were retrieved from the concurrent axillary dissection, all of which were negative for malignancy, resulting in a stage of pT4N0 [11]. All resection margins were negative.

**Figure 8:**
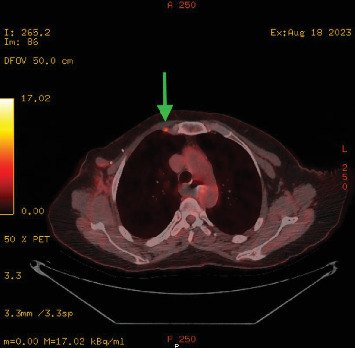
PET-CT, fused axial series, image 86 (18/08/23). Image shows abnormal internal mammary chain node (green arrow).

As complete surgical excision is curative, a repeat PET-CT was performed 1-month post-surgery to ensure all avid disease had been resected (Figure 8). PET-CT demonstrated complete excision of the FDG avid chest wall nodules and right sub-pectoral node. There was, however, interval development of mild uptake in a sub-centimetre right internal mammary node. This was deemed to be likely reactive and a short interval follow up PET CT was recommended post multi-disciplinary team meeting. A conservative approach was adopted and the patient was scheduled for clinical surveillance with haematology and breast surgery teams.

**Figure 9:**
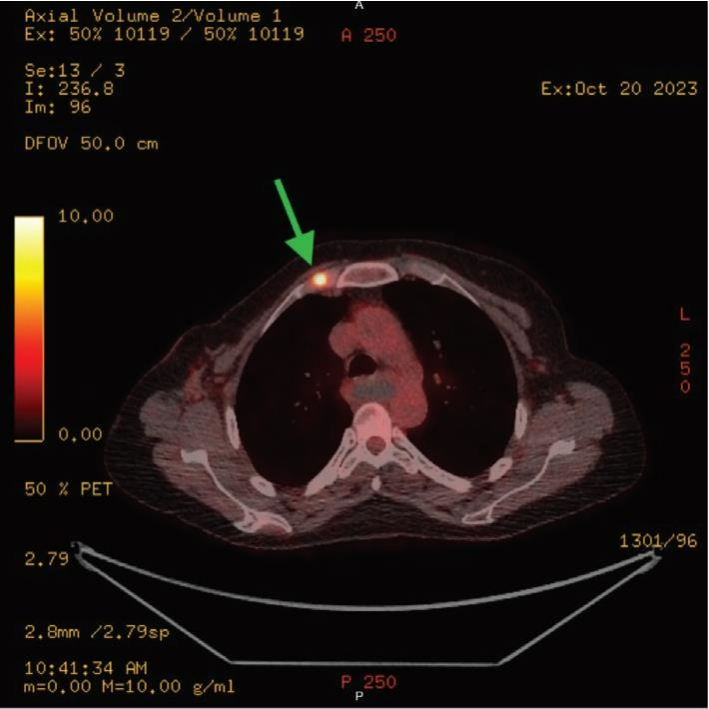
PET-CT, fused axial series, image 96 (20/10/2023). Image shows abnormal internal mammary chain node increasing in size (green arrow).

**Figure 10:**
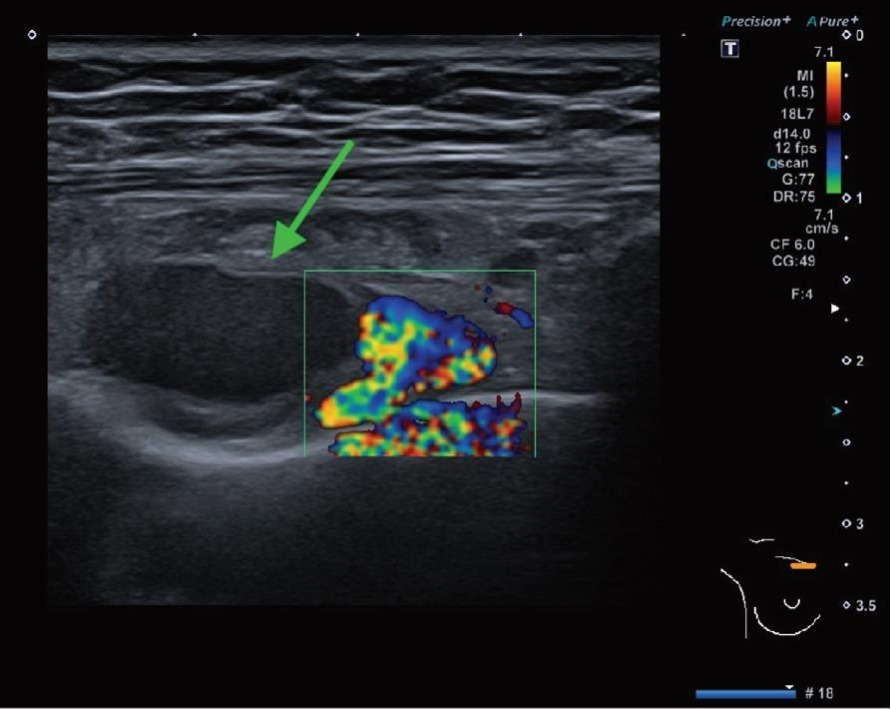
Breast ultrasound image (27/10/23) shows abnormal internal mammary chain node (green arrow).

The follow-up PET-CT showed interval increase in size in the right internal thoracic lymph node which was now intensily FDG avid and this favoured to represent a site of lymphoma rather than reactive lymphadenopathy (Figure 9). A subsequent focused ultrasound identified a 17 mm × 14 mm hypoechoic nodule lateral to the internal mammary vessels in the right second intercostal space, suspicious for malignant lymphadenopathy (Figure 10). Image guided core biopsy was performed, but histological features were suspicious but not diagnostic of lymphoma. An additional CT chest at a further 3 month interval identified a 22 mm × 15 mm enlarged right internal mammary lymph node, which had increased in size when compared to her previous PET-CT scan.

Following this, she underwent mediastinoscopy and biopsy of the right internal mammary lymph node. Histopathological examination revealed a malignant CD30 positive T-cell infiltrate, this was a similar morphology to that seen in the breast, likely representing a nodal metastasis of the BIA-ALCL.

Following MDT discussion, she was appropriately referred for urgent review by medical and radiation oncologists and commenced on chemotherapy. Patient completed six cycles of chemotherapy (BV-CHP: brentuximab vedotin, cyclophosphamide, doxorubicin, prednisone).

Complete response to the six cycles of BV-CHP was demonstrated on imaging by interval resolution of the previously noted FDG avid right internal mammary chain nodes on follow up PET-CT and treatment was well tolerated without any major toxicity. To date, after two and a half years since first presentation, the patient remains in complete clinical and radiological remission. Patient will continue undergoing regular surveillance with haematology and breast surgery teams to complete a minimum of 5 years duration of follow-up, and patient will be considered cured at this point if no evidence of relapse. In addition, patient will also continue undergoing annual surveillance mammograms until the age of 70.

## 3. Discussion

BIA-ALCL is an uncommon malignancy that poses an evolving challenge due to the increased use of implants for reconstructive and cosmetic procedures [5]. It can be considered a type of iatrogenic malignancy (non-Hodgkin's T-cell lymphoma) associated with breast surgery involving textured surface breast implants and tissue expanders [1].

The most common clinical presentation in approximately 80% of patients is of a rapidly evolving large peri-implant effusion at least 1-year post implant placement. Palpable masses, capsular contracture and lymphadenopathy are also common symptoms [1, 5]. Some patients may present with more advanced features, such as chest wall invasion, bilateral disease, lymph node involvement and distant metastases [1]. In our case study, presentation was of a rapidly developing palpable breast lump that appeared to be attached to the chest wall. This warranted an urgent referral from primary care to the symptomatic breast centre to exclude invasive carcinoma of the breast. Other differential diagnosis of BIA-ALCL would include implant rupture, infection, and systemic ALCL.

When BIA-ALCL is suspected, breast imaging including breast ultrasound plays a key role in the diagnosis as it facilitates core biopsy/fluid aspiration for histological/cytological analysis [1, 5, 12]. CD30 immunohistochemistry is characteristically positive in ALCL and may highlight small foci of lymphoma that are subtle on routine staining and its use is crucial to avoid a false benign report [1]. A post-contrast MRI breast should be performed for local staging of the disease and FDG PET-CT for distant metastases [1, 5, 12, 13].

Surgery alone with negative margins is curative in 80% of patients who present with localised disease. This requires oncologic en bloc resection with implant removal, total capsulectomy and complete tumour excision with negative margins [1, 5, 13]. Adjuvant therapy, such as chest wall radiation, targeted immune therapy, systemic chemotherapy and/or stem cell transplant, might be required when there is residual disease or invasion beyond the capsule [1, 5, 13]. Adjuvant therapy was not initially planned for our patient, however, following surveillance imaging identifying loco-regional recurrence of disease, BV-CHP chemotherapy was commenced, resulting in complete response on follow-up imaging. Sibon et al. [14] demonstrated that treatment with BV-CH(E)P resulted in improvement in progression-free survival and overall survival in comparison to CHOP/CHOP-like regimen in those with capsular infiltration [14].

After primary treatment, clinical and radiological follow-up is recommended with PET-CT every 3–6 months for the first 2 years, with annual surveillance subsequently [13]. Our case study highlights the importance of surveillance with PET-CT to ensure early detection of recurrence.

Furthermore, in Ireland, the Health Products Regulatory Authority (HPRA) encourages the public, patients and healthcare professionals to report complications associated with breast implants [15]. A small number of incident reports of suspected or confirmed cases of BIA-ALCL associated with Allergan's BIOCELL textured implants were reported; however, the specific numbers are unfortunately not publicly released due to potential patient identification [16]. A survey conducted by The European Association of Societies of Aesthetic Plastic Surgery E(A)SAPS, published in 2021, highlighted a rising trend in BIA-ALCL diagnoses across Europe, with cases increasing from 305 in 2019 to 434 in 2020 [17]. Ireland reported one case of BIA-ALCL in that study, which corresponded to an estimated prevalence of 16.5 cases per million women at risk [17]. This upward trend highlights the critical need for robust monitoring systems. The 2022 World Consensus Conference on BIA-ALCL stated that this uncommon condition is largely underreported and recommends mandatory implant registries and actions by regulatory bodies [18]. Along the same lines, the American Association of Plastic Surgeons Consensus on BIA-ALCL, published in September 2024, emphasises the need for surgeons to engage in prospective registries to provide implementable insights into these evolving diseases [19]. Within this context, the Irish Breast Implant Registry (IBIR) development project commenced in November 2023 [20], which will collect and record prospective data on breast implant and device surgeries on commencement of the registry. The IBIR aims to enhance patient safety and the quality of care by systematically tracking breast implant procedures and associated complications, including serious conditions such as BIA-ALCL, as well as collaborating with international breast implant registries, to support research aimed at improving the understanding of risk factors linked to breast implants and related devices [20].

In conclusion, survival rates for patients with BIA ALCL are >90% in early stages [1]. Multidisciplinary approach for workup and management of the disease is paramount. Patient education, awareness and vigilance are crucial for timely diagnosis. Complete surgical resection is essential for good prognosis, and clinical and radiological surveillance are required for detection of early recurrence so that adjuvant therapy may be commenced in a timely manner to improve outcomes in these patients. In addition, the establishment of national and international registries for complications related to breast implant surgery is essential to ensuring high-quality patient care, advancing the understanding of associated risk factors, and facilitating research in the field.

## Data Availability

Data sharing is not applicable to this article as no new data were created or analysed in this study.
